# Prognostic Nutritional Index Predicts Response and Prognosis in Cancer Patients Treated With Immune Checkpoint Inhibitors: A Systematic Review and Meta-Analysis

**DOI:** 10.3389/fnut.2022.823087

**Published:** 2022-07-22

**Authors:** Liwei Ni, Jing Huang, Jiyuan Ding, Junyan Kou, Tingting Shao, Jun Li, Liujie Gao, Wanzhen Zheng, Zhen Wu

**Affiliations:** Department of Oncology, Hangzhou Cancer Hospital, Hangzhou, China

**Keywords:** prognostic nutritional index, immune checkpoint inhibitor, meta-analysis, cancer, prognosis carcinoma

## Abstract

**Objective:**

To investigate the association between pretreatment prognostic nutritional index (PNI) and clinical survival outcomes for advanced-stage cancer patients treated with immune checkpoint inhibitors (ICIs).

**Methods:**

We conducted a comprehensive literature search to identify eligible studies concerning the relationship between pretreatment PNI and survival outcomes in advanced cancer patients treated with ICIs. Published data were extracted and pooled odds ratio (pOR) for objective response rate (ORR), disease control rate (DCR), and pooled hazard ratio (pHR) for overall survival (OS), progressive-free survival (PFS), along with 95% confidence intervals (95% CIs) were calculated.

**Results:**

Twelve studies with 1,359 participants were included in our study. A higher level of PNI indicated a greater ORR (pOR = 2.17, 95% CI = 1.52–3.10) and favorable DCR (pOR = 2.48, 95% CI = 1.87–3.29). Low PNI was associated with a shorter OS (pHR = 2.24, 95% CI = 1.57–3.20) and unfavorable PFS (pHR = 1.61, 95% CI = 1.37–1.88).

**Conclusion:**

Low PNI might be an effective biomarker of poor tumor response and adverse prognosis of advanced cancer patients with ICIs. Further studies are needed to verify the prognostic value of PNI in clinical practice.

## Introduction

Cancer is one of the most widespread diseases with high morbidity, mortality, and economic burden worldwide (1). Due to the development of comprehensive therapy for cancer, the cancer mortality rate fell continuously from 1997 through 2017, resulting in an overall decline of 29% in the United States (2). Immune checkpoint inhibitors (ICIs), including antibodies against cytotoxic T lymphocyte antigen-4 (CTLA-4) and programmed cell death protein 1 (PD-1) and its ligand PD-L1, can reactivate T-cell antitumor function and interfere with tumor immune escape (3, 4). Nowadays, ICIs are widely used to treat different types of malignancies and possesses a clinical curative effect on selected individuals. Therefore, identifying dependable biomarkers is required for the development of individualized treatments for candidates treated with ICIs.

Survival of individuals treated with ICIs depends on prognostic factors, such as performance status (5), body mass index (6), tumor diameter (7), PD-L1 (8), tumor mutation burden (TMB) (9), microsatellite instability (MSI), mismatch-repair deficiency (dMMR) (10), tumor-infiltrating lymphocytes (TIL) (11). Moreover, the prognostic role of several inflammation-related plasma biomarkers has been reported, including the neutrophil-to-lymphocyte ratio (NLR) (12), platelet-to-lymphocyte ratio (PLR) (13), and prognostic nutritional index (PNI).

PNI is calculated using the following formula: 10 × serum albumin (S-Alb) concentration + 0.005 × total lymphocyte count (TLC) (14). This indicator has been used for assessing the nutritional and immunological status of cancer patients (15). Nutrition and immune status have been found to be related with the efficacy of immunotherapy and the long-term outcomes of malignancies (16, 17). Peng et al. revealed that a higher level of PNI was associated with better survival outcomes in advanced non-small cell lung cancer (NSCLC) treated with ICIs (18). Kim et al. identified that PNI greater than the cutoff was correlated with favorable progressive-free survival (PFS) and overall survival (OS) in advanced esophageal squamous cell cancer (ESCC) with immunotherapy (19). A study by Zaitsu et al. showed that PNI was not a significant prognostic factor of immunotherapy in patients with lung cancer (20). Overall, the association between PNI and survival outcomes in cancer patients with immunotherapy remains obscure.

Hence, we systematically reviewed publications on the relationship between PNI and the prognoses of malignancy tumors and performed this meta-analysis to demonstrate the predictive effect of pretreatment PNI on the PFS and OS of cancer patients treated with ICIs. Additionally, we intended to evaluate the impact of PNI on ICI response in cancer patients.

## Materials and Methods

### Literature Search

A systematic search was performed to identify relevant studies from the PubMed, EMBASE, and ISI Web of Science. The following key words were used: “prognostic nutritional index” (OR “PNI”) AND “cancer” (OR “carcinoma”) AND “immunotherapy” (OR “immune checkpoint inhibitor” OR “PD-L1 inhibitor” OR“PD-1 inhibitor”). The search was updated in June 2021. In addition, a manual search in the reference lists was carried out to screen other potential eligible publications. The entire search process was conducted independently by two authors (Wanzhen Zheng and Jiyuan Ding), and a third person (Jun Li) was invited to settle any disagreements. This study was performed following the Preferred Reporting Items for Systematic Reviews and Meta-Analyses (PRISMA) guidelines (21).

### Study Selection

Eligible publications must meet the following criteria: (1) the full text must be searchable in English; (2) investigation of the prognostic value of PNI in advanced cancer patients treated with immunotherapy; (3) PNI were calculated in cancer patients prior to immunotherapy; (4) the cutoff values of pretreatment PNI were obtainable; (5) available data for calculating survival estimates, such as odds ratio (OR) or hazard ratio (HR) with 95% confidence intervals (CIs). Abstracts, meetings, case reports, reviews, editorials, and laboratory studies were excluded. Two authors (Liujie Gao and Tingting Shao) screened the original studies independently and reached a consensus in the included studies. Cohen’s kappa statistic was applied to measure chance−corrected agreement between reviewers (SPSS version 22.0, SPSS Inc., Chicago, IL, United States).

### Data Extraction and Quality Assessment

We extracted the data of each individual study including the following variables: last name of first author, publication year, country or region, study duration, study design, sample size, age, performance status, tumor type, stage, treatment methods, the proportion of first-line treatment with immunotherapy, cutoff values of PNI, HRs with 95%CIs for OS and PFS. HRs in multivariable analyses were preferentially extracted. ORs with 95%CIs for objective response rate (ORR), disease control rate (DCR), and immune-related adverse events (irAEs) were also extracted if available. The Newcastle–Ottawa Scale (NOS, scores of 0–9 stars) was applied to assess the quality of enrolled publications. An article with NOS scores ≥ 7 were regarded as a high-quality studies. Two reviewers assessed each study independently and reached a consensus after discussion.

### Statistical Analysis

Each HR or OR in original study was extracted. The primary outcomes were reported as pooled HRs (pHRs) with 95% CIs for OS and PFS. The secondary outcomes were pooled ORs (pORs) with 95% CIs for ORR, DCR and irAEs. Estimates were first summarized using the fixed-effects model to identify heterogeneity. If heterogeneity was significant, the random-effects model was eventually applied (22). The Chi-square test and *I*^2^ statistic were used to assess the statistical heterogeneity among articles. *P* < 0.05 being considered statistically significant and *I*^2^ > 50% indicating higher heterogeneity. Subgroup analyses and sensitivity analyses were conducted to elaborate on the prognostic value of PNI on survival outcomes for cancer patients with immunotherapy or to reduce and explain the statistical heterogeneity if necessary. Publication bias was visually inspected in graphical funnel plots and quantitatively evaluated by Egger’s test (23, 24). If not directly available, ORs with 95%CIs were calculated in a 2-by-2 contingency table using data obtained from the original studies (25). All tests were two-tailed and a *P*-value less than 0.05 was considered statistically significant. Statistical analyses were conducted by using Stata 14.0 (Stata Corporation, College Station, TX, United States).

## Results

### Literature Search

**FIGURE 1:**
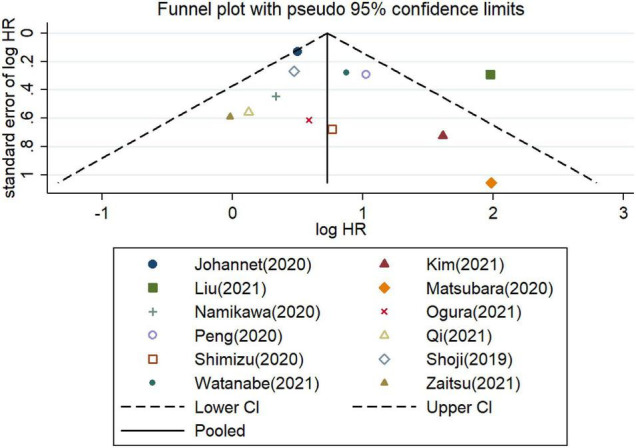
The literature search process.

The flowchart of literature search illustrates the selection process (Figure 1). Initially, 149 relevant publications were screened after searching for PubMed, EMBASE, and ISI Web of Science. Then, 85 duplicate records were identified and omitted. After reviewing titles and abstracts, the remaining 21 studies were reviewed in detail. After excluding 9 papers that lacked data on OS and PFS or did not calculate the pretreatment PNI, we finally included 12 articles in this meta-analysis. The kappa statistic indicated a satisfactory interrater agreement between two reviewers (kappa = 0.88).

### Study Characteristics and Quality Assessment

The main characteristics of the eligible articles are depicted in Table 1. The period of these studies was in the range of 2012–2020, with the included studies being published from 2019 to 2021. Twelve cohort studies consisted of 1,359 cancer patients with immunotherapy. Eleven of the 12 studies were from Asian countries (7 from Japan, 3 from China, and 1 from Korea). The cut-off value of PNI ranged from 31.1 to 46.05, with the value being 40 and 45 in 3 studies, respectively. All of these studies reported HRs for OS (8 in multivariate analysis and 4 in univariate analysis), and 10 of them reported HRs for PFS (7 in multivariate analysis and 3 in univariate analysis). PNI was identified as a significant independent predictor in 41.7% (5/12) of the studies on OS and in 50.0% (5/10) of the studies on PFS. Additionally, the NOS scores of 12 included articles were ≥ 7, thus indicating high quality.

**TABLE 1:** Characteristics of included studies for meta-analysis.

References, region	Study design	Study duration	Number	Age	Performance status	Tumor type	Stage	Treatment	Proportion of first line treatment	Cut-off value for PNI	Survival data (HRs and 95%CIs)	NOS scores
Watanabe et al. (28), Japan	Retrospective	2015–2019	110	NA	0–3	GC or GOC	IV	Nivolumab	NA	40^#^	OS, 2.40 (1.38–4.15) in MVA; PFS, 1.56 (0.95–2.56) in MVA	8
Ogura et al. (31), Japan	Retrospective	2019–2020	34	Median, 72	0–2	NSCLC	III-IV	Pembrolizumab, atezolizumab + CT	100%	40^#^	OS, 1.80 (0.54–5.98) in UVA; PFS, 1.59 (0.57–4.38) in UVA	7
Qi et al. (32), China	Prospective	2015–2020	53	Median, 65	0–2	SCLC	IV	Atezolizumab + CT	100%	48^##^	OS, 1.13 (0.38–3.36) in MVA;	7
Zaitsu et al. (20), Japan	Retrospective	2016–2020	95	Mean, 70.9	0–4	Lung cancer	III-IV	Nivolumab, pembrolizumab, atezolizumab	First-line (36%)	43^#^	PFS, 0.93 (0.38–2.24) in MVA; OS, 0.98 (0.31–3.13) in MVA	8
Liu et al. (27), China	Retrospective	2018–2019	123	Mean, 59.9	0–2	NSCLC	IIIB-IV	Nivolumab, pembrolizumab sintilimab, Camrelizumab, toripalimab ± CT	42.3%	46.05^##^	PFS, 2.698 (1.752–4.153) in MVA; OS, 7.222 (4.081–12.781) in MVA	8
Kim et al. (19), Korea	Retrospective	2015–2019	60	Median, 68	0–2	ESCC	III-IV	Nivolumab, pembrolizumab	0%	35.93^##^	PFS, 4.07 (1.29–12.90) in MVA; OS, 5.02 (1.21–20.76) in MVA	8
Shimizu et al. (33), Japan	Retrospective	2017–2019	27	Median,73	0–4	UC	IV	Pembrolizumab	100%	45^##^	PFS, 2.10 (0.75–5.93) in VVA; OS, 2.15 (0.57–8.11) in MVA	7
Peng et al. (18), China	Retrospective	2017–2019	102	Median, 62	0–2	NSCLC	IIIB-IV	Nivolumab, pembrolizumab, toripalimab, sintilimab	18.6%	45^#^	PFS,1.92 (1.14–3.25) in MVA; OS,2.79 (1.57–4.95) in MVA	8
Namikawa et al. (30), Japan	Retrospective	2017–2019	27	Median, 71	0–2	GC	III-IV	Nivolumab	50%	31.1^###^	PFS,1.18 (0.51–2.74) in UVA; OS,1.39 (0.58–3.34) in MVA	7
Matsubara et al. (29), Japan	Retrospective	2018–2019	24	Median, 64.5	0–2	NSCLC	NR	Atezolizumab	0%	40^#^	OS,7.28 (0.92–57.4) in UVA	7
Johannet et al. (26), America	Retrospective	2012–2020	629	Mean, 63	0–4	Several types of cancer	III-IV	Atezolizumab, avelumab, durvalumab, ipilimumab, nivolumab, pembrolizumab, tremelimumab ± CT	71.2%	45^#^	PFS,1.34 (1.06–1.69) in MVA; OS,1.65 (1.27–2.13) in MVA	8
Shoji et al. (34), Japan	Retrospective	2015–2019	102	Mean, 69	0–4	NSCLC	III-IV	Nivolumab, atezolizumab, pembrolizumab ± CT	19.6%	45.5^##^	PFS,1.704 (1.04–2.83) in MVA; OS,1.61 (0.95–2.75) in MVA	8

*NA, not available; NSCLC, non-small cell lung cancer; UVA, univariate analysis; MVA, multivariate analysis; GC, Gastric; GOC, Gastro-esophageal Junction Cancer; ESCC, esophageal squamous cell carcinoma; UC, urothelial carcinoma; PNI, prognostic nutritional index; CT, chemotherapy; PFS, progression-free survival; OS, overall survival.^#^The cutoff values for PNI were used according to previous reports. ^##^The cutoff values for PNI were determined according to receiver operating characteristic analysis. ^###^The median of PNI was used for a cutoff value.*

### Correlation Between Pretreatment Prognostic Nutritional Index and Patient Survival

**FIGURE 2:**
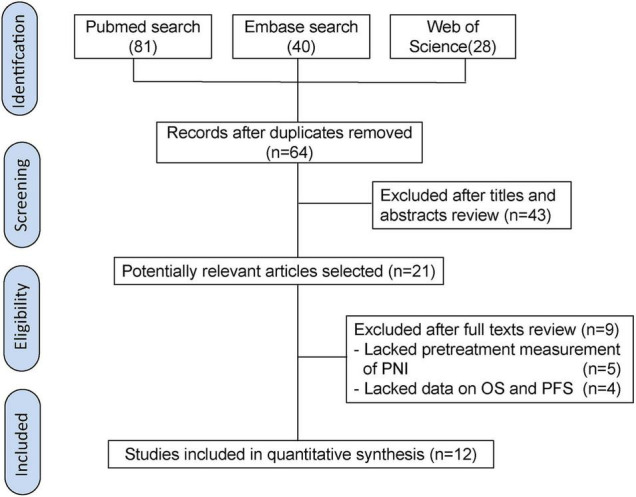
Meta-analysis of impact of PNI on overall survival of patients treated with immune checkpoint inhibitors.

Five observational studies reported that PNI was an independent indicator for a shortened OS in cancer patients with immunotherapy (18, 19, 26–28), while PNI was not considered as a prognostic factor for OS in seven studies (20, 29–34). Pooled analysis of all selected publications revealed that the PNI values higher than the cutoff value predicted a poor OS (pHR = 2.24, 95% CI = 1.57–3.20, Figure 2) in advanced cancer patients treated with immune checkpoint inhibitors.

**FIGURE 3:**
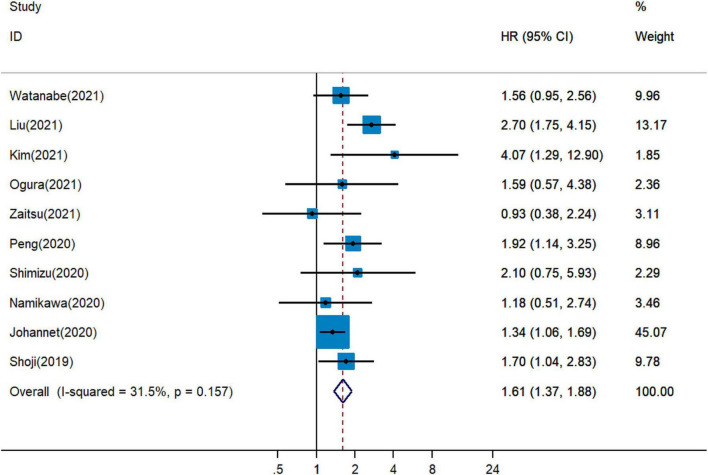
Meta-analysis of impact of PNI on progressive-free survival of patients treated with immune checkpoint inhibitors.

Five publications showed that PNI was an independent predictor of unfavorable PFS in cancer patients with immunotherapy (18, 19, 26, 27, 34), whereas five cohort studies detected no significant relationship between PNI and PFS (20, 28, 30, 31, 33). The combined analysis of ten studies that enrolled 1,282 participants showed that a higher PNI level was associated with worse PFS (pHR = 1.61, 95% CI = 1.37–1.88, Figure 3).

### Correlation Between Pretreatment Prognostic Nutritional Index and Immune Checkpoint Inhibitor Response

**FIGURE 4:**
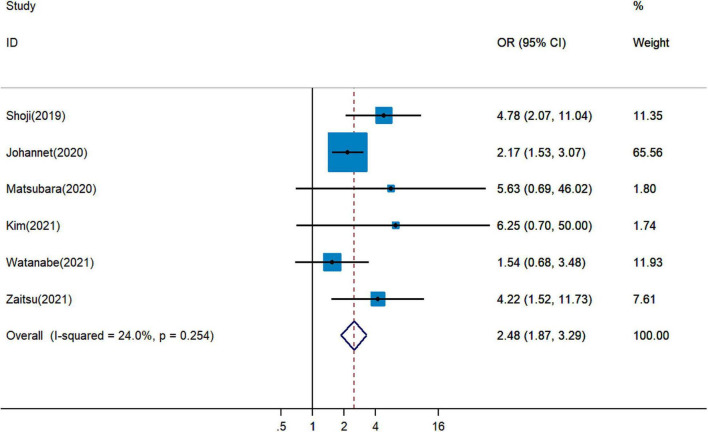
Meta-analysis of impact of PNI on disease control rate of patients treated with immune checkpoint inhibitors.

**FIGURE 5:**
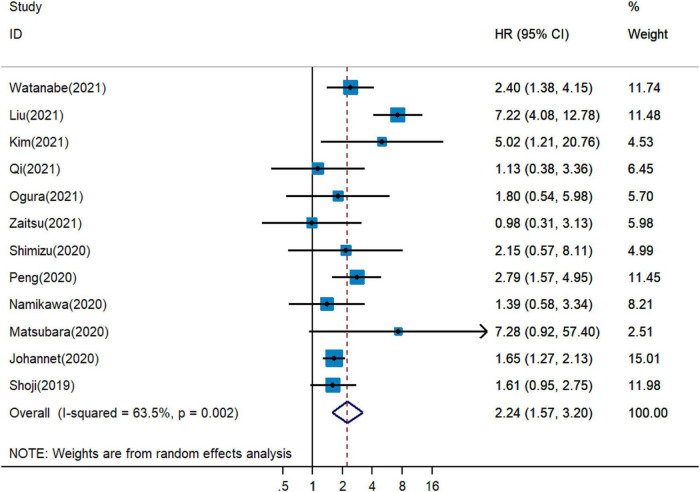
Meta-analysis of impact of PNI on objective response rate of patients treated with immune checkpoint inhibitors.

It remained uncertain among six cohort studies on the association between pretreatment PNI and DCR for cancer patients treated with ICIs. Pooled analysis revealed that a higher PNI level was associated with greater DCR (pOR = 2.48, 95% CI = 1.87–3.29, Figure 4) (19, 20, 26, 28, 29, 34). The combined analysis of three studies on the association between PNI and ORR showed that PNI higher than the cutoff indicated more favorable ORR (pOR = 2.17, 95% CI = 1.52–3.10, Figure 5) (26, 28, 34).

### Correlation Between Pretreatment Prognostic Nutritional Index and Immune-Related Adverse Events

**FIGURE 6:**
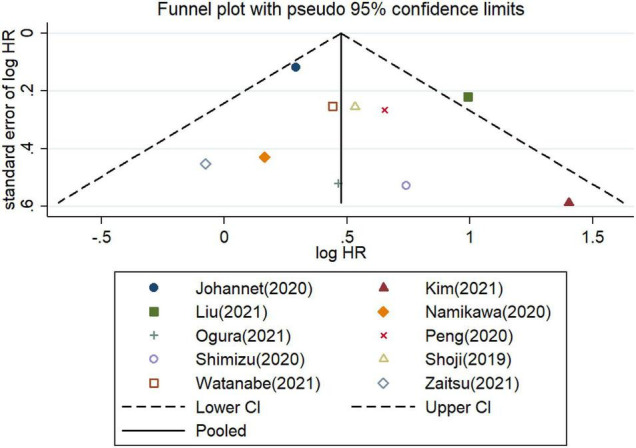
Meta-analysis of impact of PNI on immune-related adverse events in patients treated with immune checkpoint inhibitors.

Paradoxical results involved two studies on the association between pretreatment PNI and irAEs for cancer patients treated with ICIs. Pooled analysis revealed that a higher PNI level might be associated with more irAEs (pOR = 2.42, 95% CI = 0.58–10.11, Figure 6) (18, 27), but with no statistical significance.

### Heterogeneity and Subgroup Analysis

Considering the substantial heterogeneity, we applied the random-effects model for pooled analysis of HRs of OS (*I*^2^ = 63.5%, *P* = 0.002, Figure 2). We further conducted subgroup analyses to detect the source of heterogeneity. When stratified on basis of the median cutoff values, the pHRs of OS for PNI ≤ 40 (pHR = 2.29, 95% CI = 1.52–3.44) and for PNI > 40 (pHR = 2.14, 95% CI = 1.30–3.53) were analogous to the overall estimate of subgroups (Supplementary Figure 1). *I*^2^ decreased to 0.0% in PNI ≤ 40 group and it slightly increased to 63.5% in PNI > 40 group (Supplementary Figure 1). Based on the analysis method, the pHRs of OS for PNI in univariate analysis (pHR = 2.16, 95% CI = 1.34−3.49) and in multivariate analysis (pHR = 2.45, 95% CI = 1.59–3.77) were similar to the combined estimate (Supplementary Figure 2). *I*^2^ decreased to 0.0% in univariate analysis and it increased to 73.7% in multivariate analysis (Supplementary Figure 2). The results of subgroup analysis stratified by the country were unstable with *I*^2^ increased to 81.4% in China group and it decreased in Japan and in other country groups (Supplementary Figure 3). Based on the cancer type, the pHRs of OS for PNI in gastrointestinal cancer (pHR = 2.24, 95% CI = 1.33−3.78), in lung cancer (pHR = 2.36, 95% CI = 1.28–4.35) and in other types of cancer (pHR = 1.67, 95% CI = 1.29−2.15) were analogous to the overall estimate (Supplementary Figure 4).

Considering the minor heterogeneity across the studies on PFS (*I*^2^ = 31.5%, Figure 3), the fixed-effects model was applied for combined analysis of HRs. The involved studies were stratified into subgroups according to the type of cancer, country, cut-off value, and analysis method. In the subgroup analysis of studies of PNI for PFS, no significant difference was found between PNI > 40 group and PNI ≤ 40 group. The pHRs of PFS for PNI ≤ 40 and for PNI > 40 were 1.64 (95% CI = 1.13–2.38) and 1.60 (95% CI = 1.35–1.90), respectively (Supplementary Figure 5). Based on the cancer type, the pHRs of PFS for PNI in gastrointestinal cancer (pHR = 1.64, 95% CI = 1.10−2.45), in lung cancer (pHR = 1.95, 95% CI = 1.51–2.52) and in other types of cancer (pHR = 1.37, 95% CI = 1.09−1.72) were analogous to the overall estimate (Supplementary Figure 6). The results of subgroup analysis stratified by the analysis method were unstable with I2 increased to 51.4% in multivariate analysis and it decreased to 0.0% in univariate analysis (Supplementary Figure 7). When stratified by country, I2 decreased from 31.5 to 0.0% in Japan and China groups, and heterogeneity between subgroups was significant (*P* = 0.037, Supplementary Figure 8), indicating that the country increased heterogeneity in the pHR of PFS for PNI. The results demonstrated that country might be a source of heterogeneity among publications on the association between PNI and PFS.

### Sensitivity Analysis and Publication Bias

The stability of pHR for OS was evaluated by the trim-and-fill method in the random-effects model. No obvious changes were detected between the previous and new pHRs (Supplementary Figure 9). Additionally, the new results did not flip significantly after excluded the involved articles one by one. None of the included publications disturbed the stability of the overall estimate (Supplementary Figure 10). The stability of pHR for PFS was measured in the fixed-effects model. The results revealed that the new pHR was similar to the previous one (Supplementary Figure 11). Pooled estimates did not significantly change regardless of which study was excluded (Supplementary Figure 12).

**FIGURE 7:**
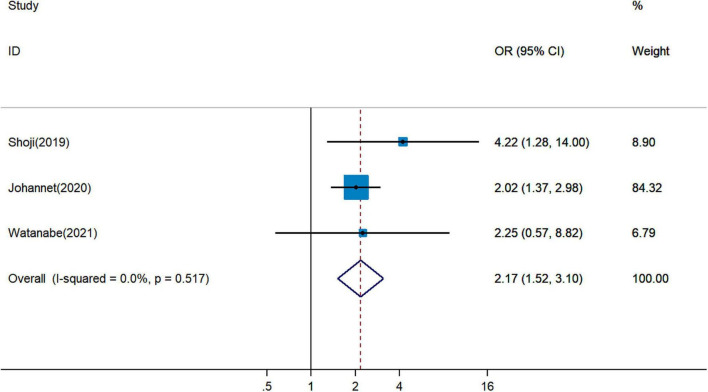
Funnel plots of studies on the association between PLR and overall survival in patients treated with immune checkpoint inhibitors.

**FIGURE 8:**
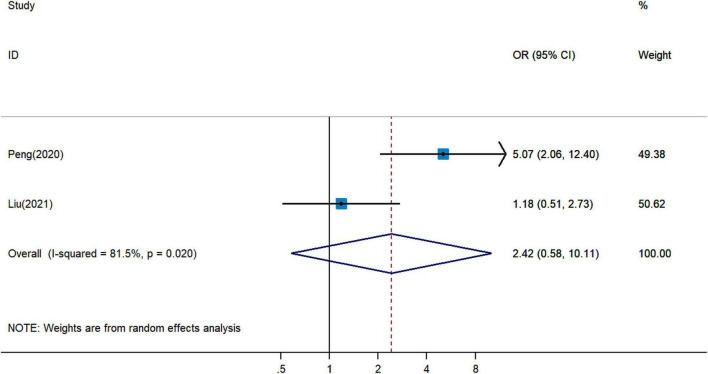
Funnel plots of studies on the association between PLR and progressive-free survival in patients treated with immune checkpoint inhibitors.

Furthermore, the potential publication bias was explored for the meta−analysis of OS and PFS. These funnel plots showed that most of the publications are approximately symmetrical, suggesting that the publication bias for OS (Figure 7) and for PFS (Figure 8) were not apparent. To further test the symmetry of graphs, we performed Egger’s test and found no significant publication bias (*P* = 0.505 for OS and *P* = 0.381 for PFS).

## Discussion

A low level of PNI is associated with unfavorable cancer prognosis, depending on the meta-analyses of various tumors, including biliary tract (35), nasopharyngeal (36), lung (37), esophageal (38), gastric (39), pancreatic (40), hepatocellular (41), and gynecological cancers (42). However, several retrospective studies demonstrated paradoxical results regarding the impact of PNI on cancer immunotherapy. Hence, we systematically review the publications on the association between PNI and the prognosis of cancer patients with immunotherapy. Pooled analysis of selected studies revealed that PNI was an independent prognostic factor for OS and PFS in patients treated with ICIs.

PNI is an immune-nutritional parameter based on S-Alb level and lymphocyte count. The mechanism by which low PNI affects ICI response and patient survival is not fully understood. Hypoalbuminemia can result in physiological dysfunctions, including loss of drug efficacy, abnormal activation of systematic inflammation, and impaired immunity (43). Turner et al. revealed that shortened OS in individuals with higher pembrolizumab clearance is associated with cancer cachexia and elevated protein turnover secondary to chronic inflammation (44). During the recruitment of inflammatory cells including lymphocytes, different types of inflammatory mediators are released, and promote tumor progression. Additionally, increasingly released endogenous steroids may dampen immune cell activity and abrogate ICI response (45). Moreover, S-Alb levels may decrease owing to malnutrition, inflammation, and the development of malignancy, especially in advanced-stage cancer patients (46). Consequently, a low S-Alb level could promote tumor development and cancer-related inflammation, which worsen prognosis. Lymphocytes are involved in regulating host immunity and specific killing of cancer cells. Lymphocyte subsets imbalance and dysfunction are associated with cancer progression (47). Yang et al. identified that CD8 (+) FoxP3 (+) regulatory T cells abrogate tumor-specific CD8 (+) cytotoxic T lymphocytes (CTLs) responses and contribute to hepatocellular cancer (HCC) immune escape and disease progression (48). Increasing the ratio of CTLs to regulatory T cells could enhance ICI responses for various kinds of malignancies (49). ICIs treatment could increase the frequency of tumor-specific CTLs in HCC patients (50). Overall, both malnutrition (hypoalbuminemia) and immune dysfunction (based on the TLC) do damage to the prognosis of cancer patients treated with ICIs.

This systematic review demonstrating that low PNI is associated with poor ICI response and adverse prognosis in advanced cancer patients. Pooled analysis overcomes the disadvantage of a single study with limited sample size. The quality of involved articles is high, with all of these studies have a high NOS score. Moreover, no significant publication bias for PFS and OS was detected across the 12 enrolled publications, which strengthens the statistical power to draw convincing conclusions. Additionally, the predictive effect of low PNI on poor prognosis has been revealed in patients with targeted therapy, such as advanced NSCLC patients treated with gefitinib or erlotinib (51), extensive-stage small-cell lung cancer patients treated with anlotinib (52), and metastatic renal cell cancer patients with sorafenib or sunitinib (53). Generally, PNI can qualify as a convenient, cost-effective, and non-invasive indicator and can be potentially applied for predicting cancer prognosis.

However, there are some limitations to this meta-analysis. First, unadjusted factors from retrospective observational studies might lead to bias in the estimation of effects. Second, the distinct cutoff values of PNI might bring about noticeable heterogeneity. Third, the cancer type and definition of tumor stage was different in the involved studies. Fourth, differences in types of ICIs and combination therapy may lead to bias. Fifth, the pooled estimate of two studies on the association between PNI and irAEs was not significant with limited statistical power. Sixth, the eligible studies were generally from Asia, especially from Japan, so it remains uncertain whether the pooled results can be extended to western populations as well. Seventh, the combined analysis of HRs in two types of analysis methods might result in a potential source of bias. These limitations could be addressed by further studies with larger and well-matched cohorts.

## Conclusion

This meta-analysis demonstrated that a low level of pretreatment PNI was a significant predictor of unfavorable response and poor prognosis in individuals treated with ICIs. As a nutritional and immunological parameter, PNI might guide the decision on nutrition treatment to adjust the patient’s nutritional status and immune function. Early and effective nutrition interventions might further contribute to survival in cancer patients treated with ICIs. Moreover, the predictive effect of PNI on the prognosis of individuals with immunotherapy need to be further assessed in prospective cohorts. More researches are needed to explore the influence of the optimal nutritional intervention on clinical efficacy of immunotherapy in cancer patients.

## Data Availability Statement

The original contributions presented in this study are included in the article/Supplementary Material, further inquiries can be directed to the corresponding author/s.

## Author Contributions

LN and ZW designed the study. WZ, JK, and JL performed the systematic search. LG and TS selected eligible articles and conducted the quality assessment. LN, ZW, and JD analyzed, interpreted the data, and drafted the manuscript. JH revised the manuscript. All authors have approved the final version of the manuscript.

## Conflict of Interest

The authors declare that the research was conducted in the absence of any commercial or financial relationships that could be construed as a potential conflict of interest.

## Publisher’s Note

All claims expressed in this article are solely those of the authors and do not necessarily represent those of their affiliated organizations, or those of the publisher, the editors and the reviewers. Any product that may be evaluated in this article, or claim that may be made by its manufacturer, is not guaranteed or endorsed by the publisher.
