# Supplementary material for: Prognostic Nutritional Index Predicts Response and Prognosis in Cancer Patients Treated With Immune Checkpoint Inhibitors: A Systematic Review and Meta-Analysis
Source: Front Nutr. 2022 Jul 22;9:823087. doi: 10.3389/fnut.2022.823087 (PMC9353139; doi:10.3389/fnut.2022.823087)
Supplement: Supplementary file 1 [file Presentation_1.pdf]

Prognostic nutritional index predicts response and prognosis in cancer patients treated with immune checkpoint inhibitors: a systematic review and meta-analysis

Liwei Ni, Jiyuan Ding, Junyan kou, Tingting Shao, Jun Li, Liujie Gao, Wanzhen Zheng, Jing huang*, Zhen Wu*
Department of Oncology, Hangzhou cancer hospital, Hangzhou, Zhejiang, 310000, P.R. China

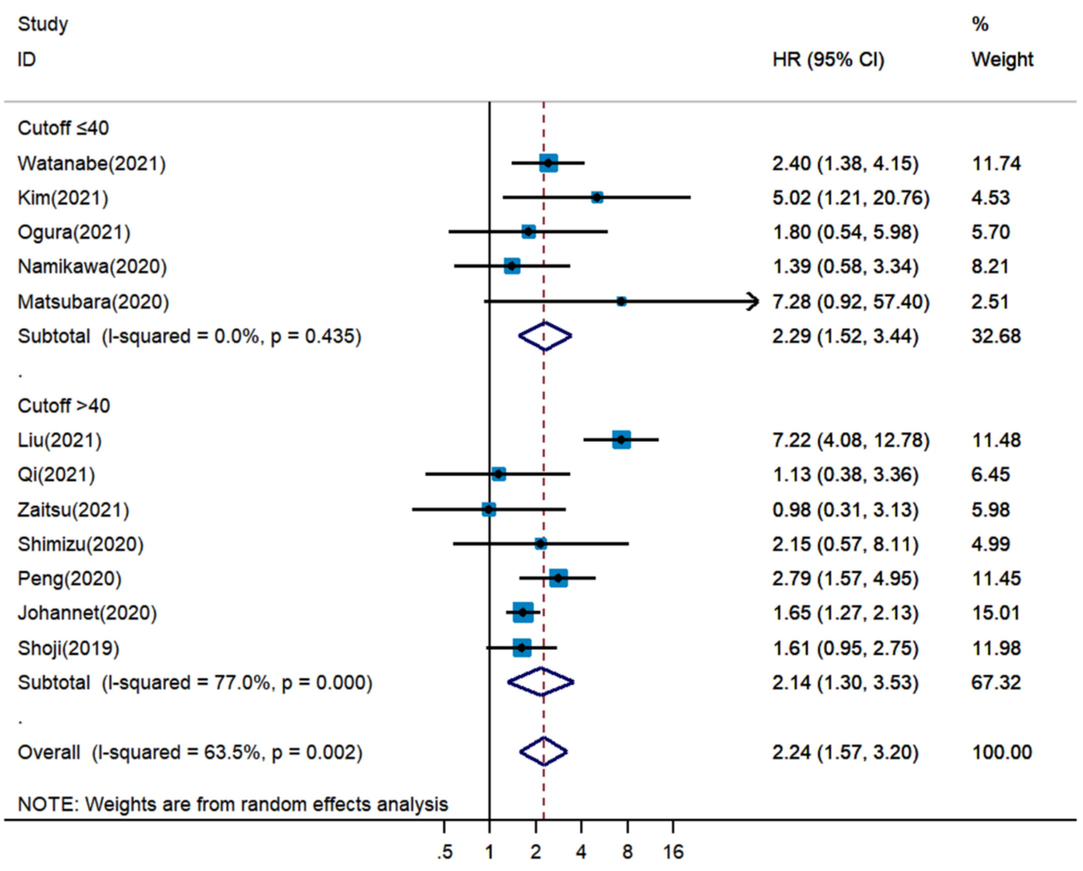

sFig.1 Meta-analysis of the association between PNI and overall survival stratified by cutoff value among patients treated with immune checkpoint inhibitors

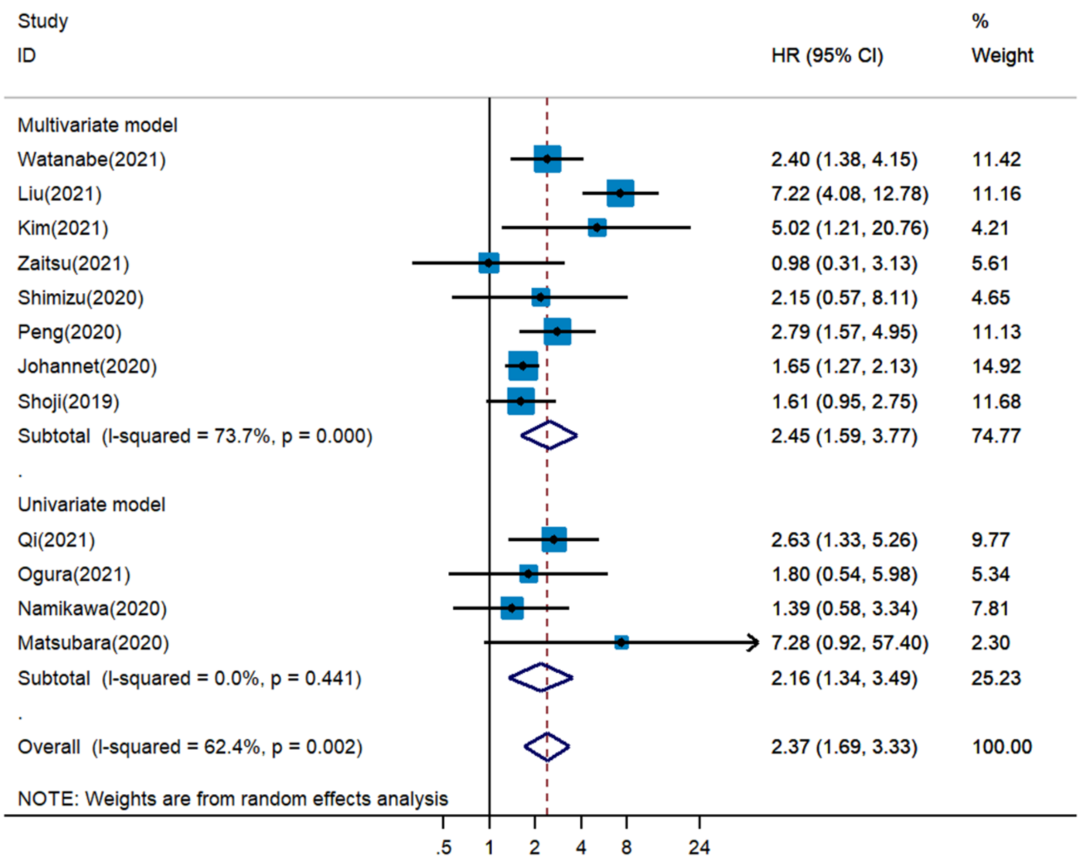

sFig.2 Meta-analysis of the association between PNI and overall survival stratified by analysis method among patients treated with immune checkpoint inhibitors

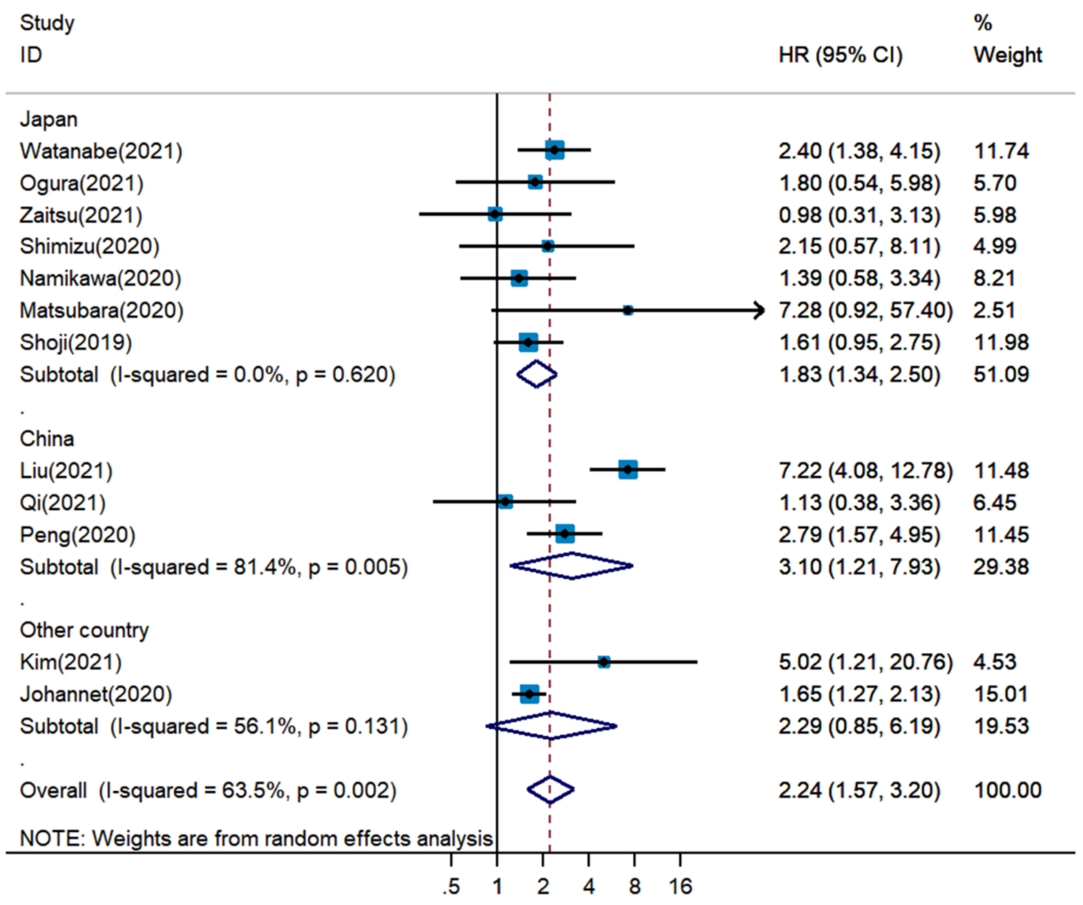

sFig.3 Meta-analysis of the association between PNI and overall survival stratified by country among patients treated with immune checkpoint inhibitors

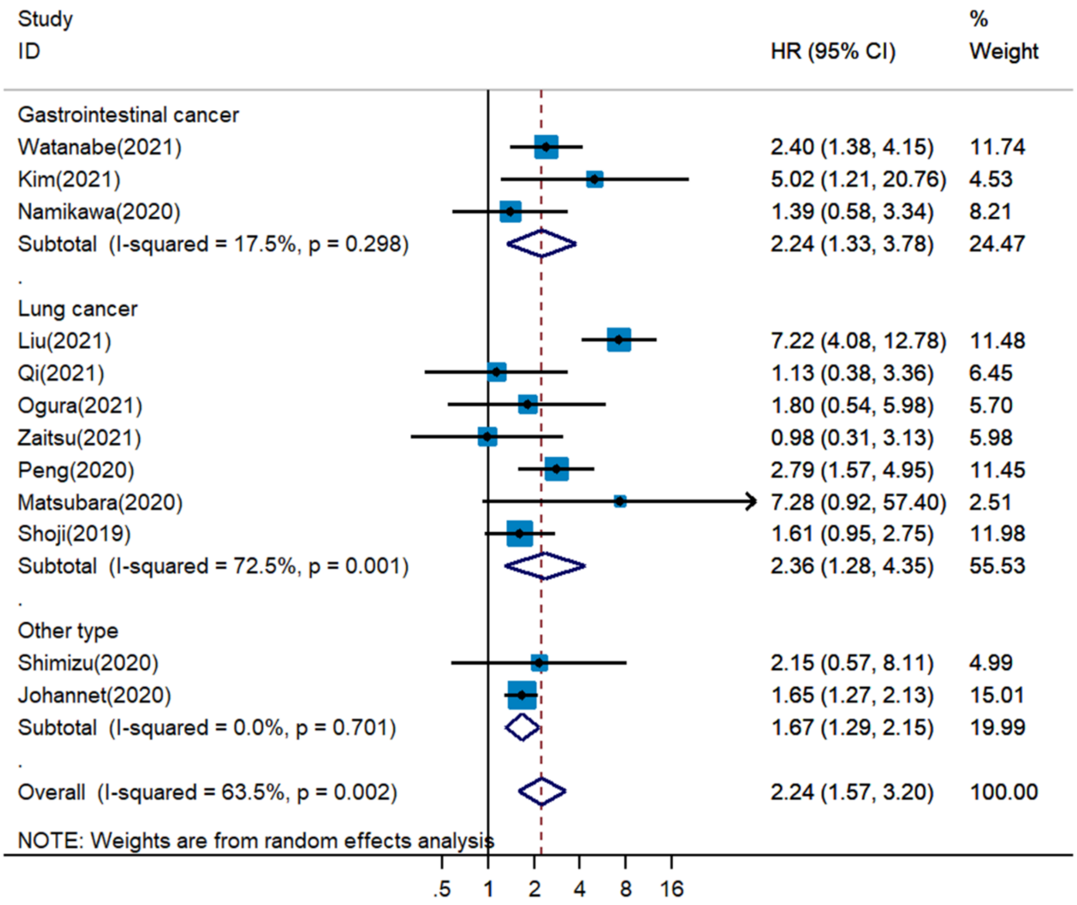

sFig.4 Meta-analysis of the association between PNI and overall survival stratified by cancer type among patients treated with immune checkpoint inhibitors

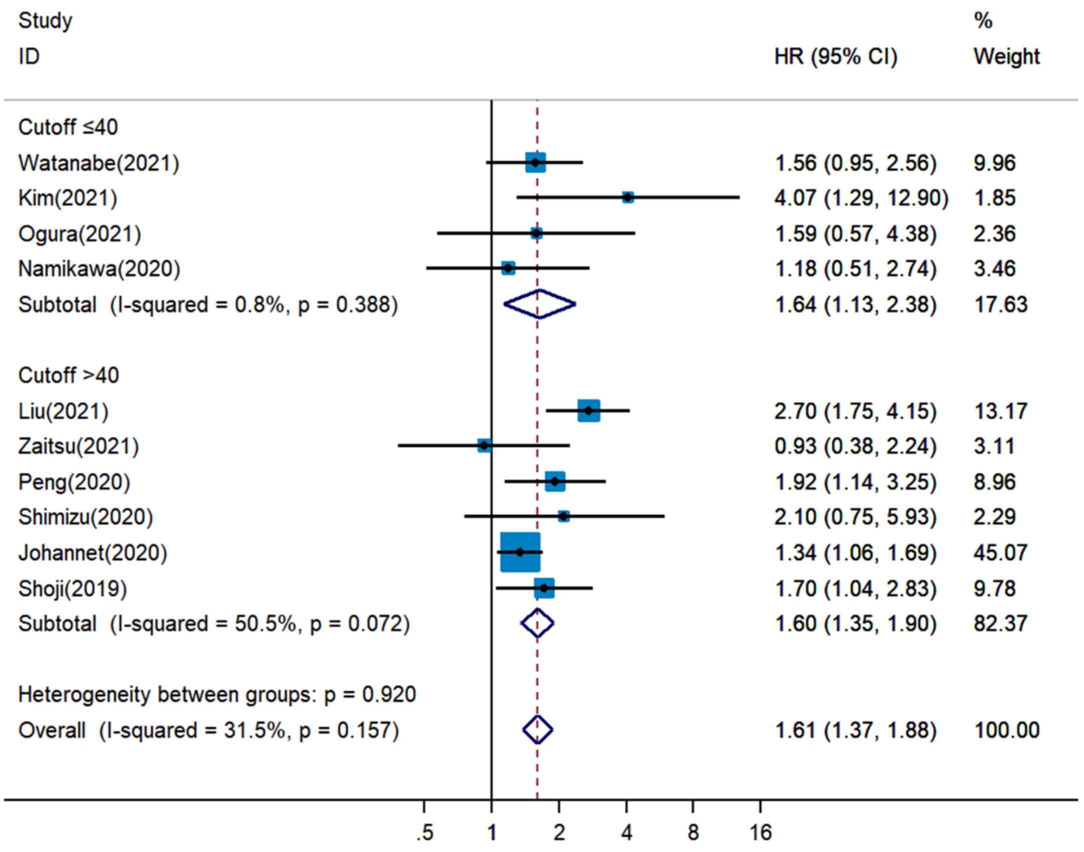

sFig.5 Meta-analysis of the association between PNI and progressive-free survival stratified by cutoff value among patients treated with immune checkpoint inhibitors

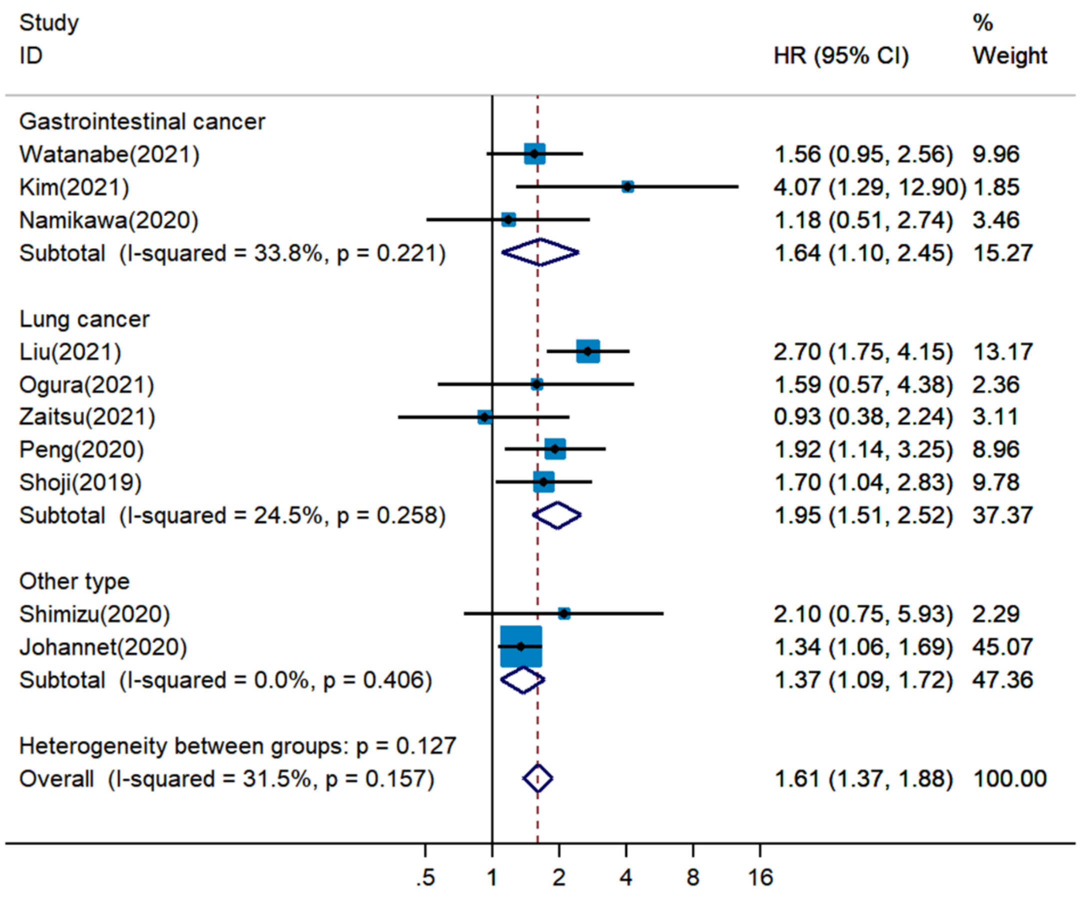

sFig.6 Meta-analysis of the association between PNI and progressive-free survival stratified by cancer type among patients treated with immune checkpoint inhibitors

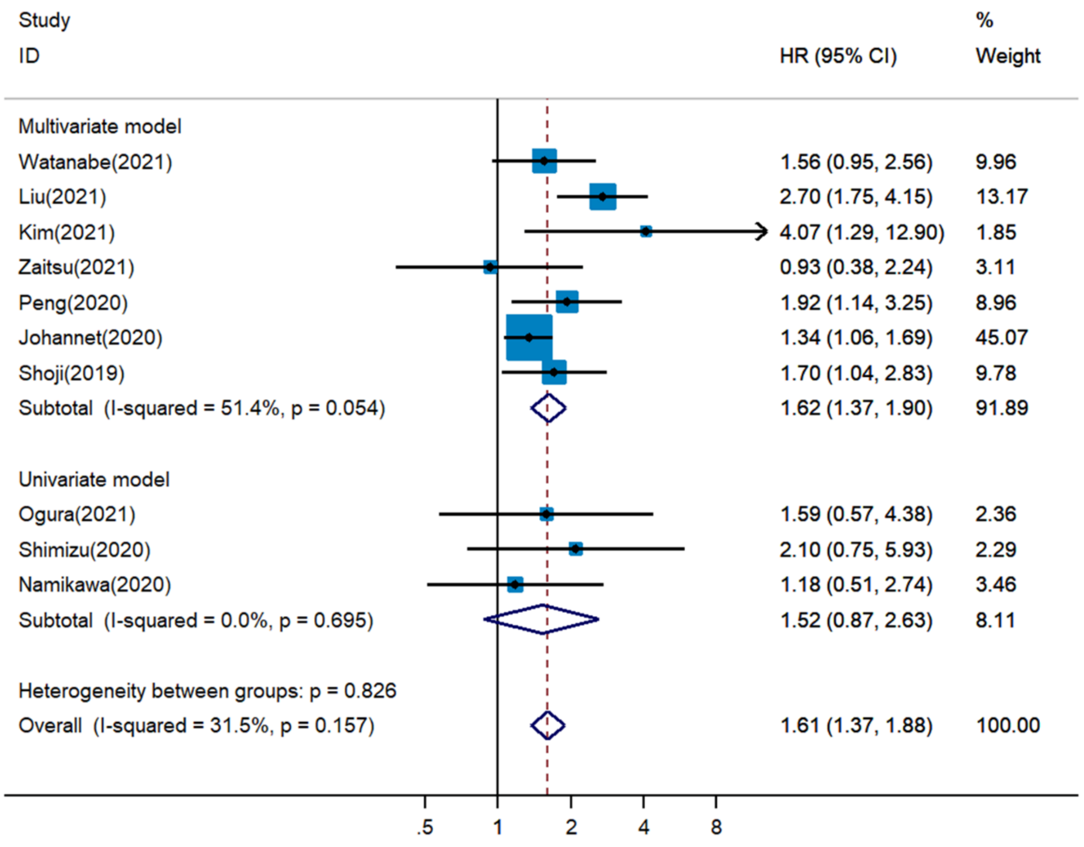

sFig.7 Meta-analysis of the association between PNI and progressive-free survival stratified by analysis method among patients treated with immune checkpoint inhibitors

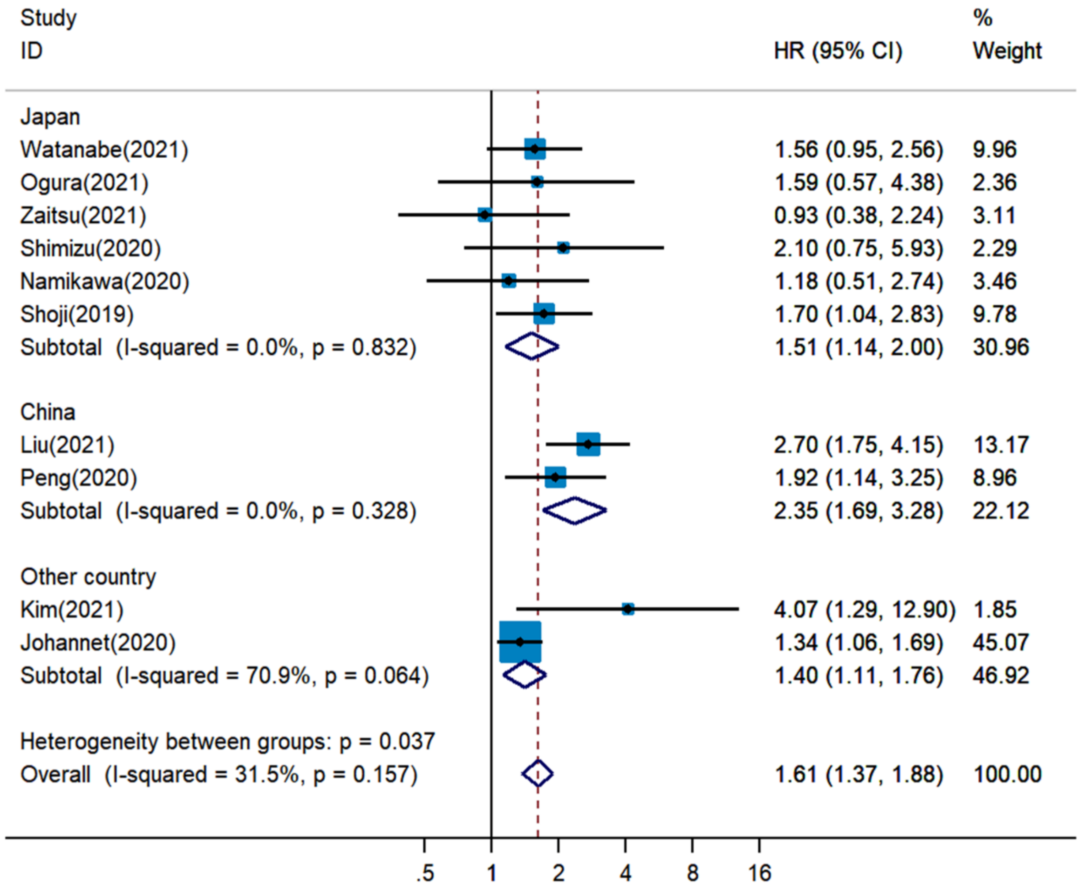

sFig.8 Meta-analysis of the association between PNI and progressive-free survival stratified by country among patients treated with immune checkpoint inhibitors

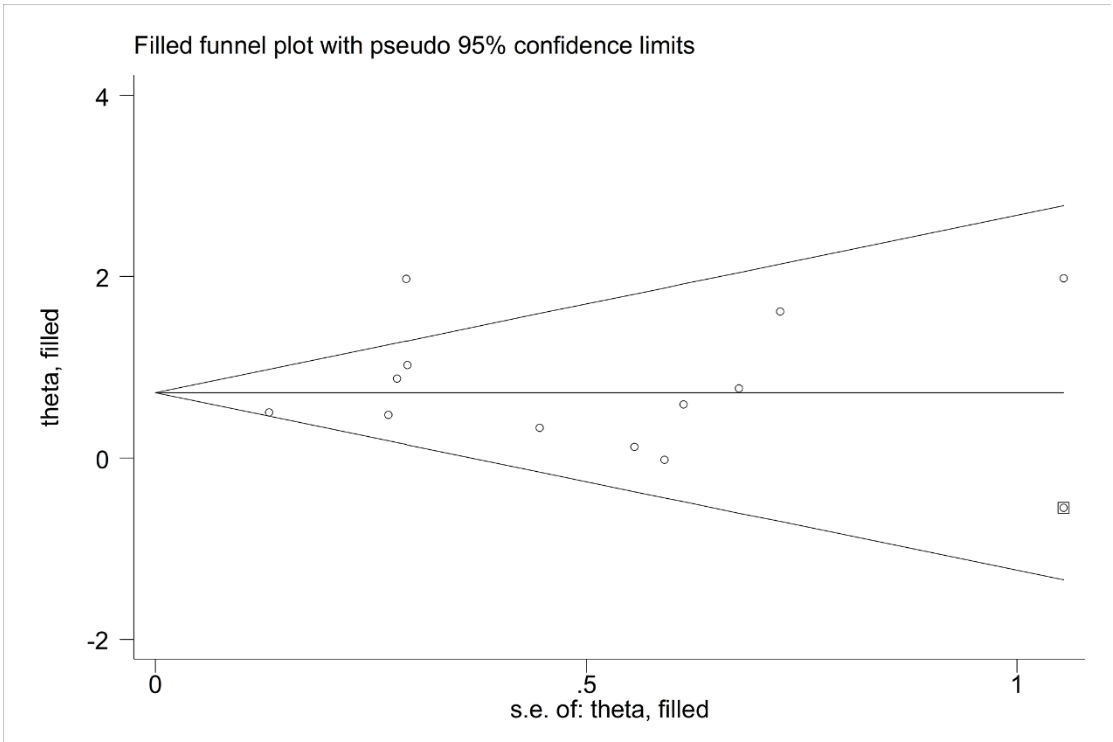

sFig.9 Metatrim test of studies included in meta-analysis of PNI for OS (new pHR = 2.169, 95%CI = 1.524−3.086)

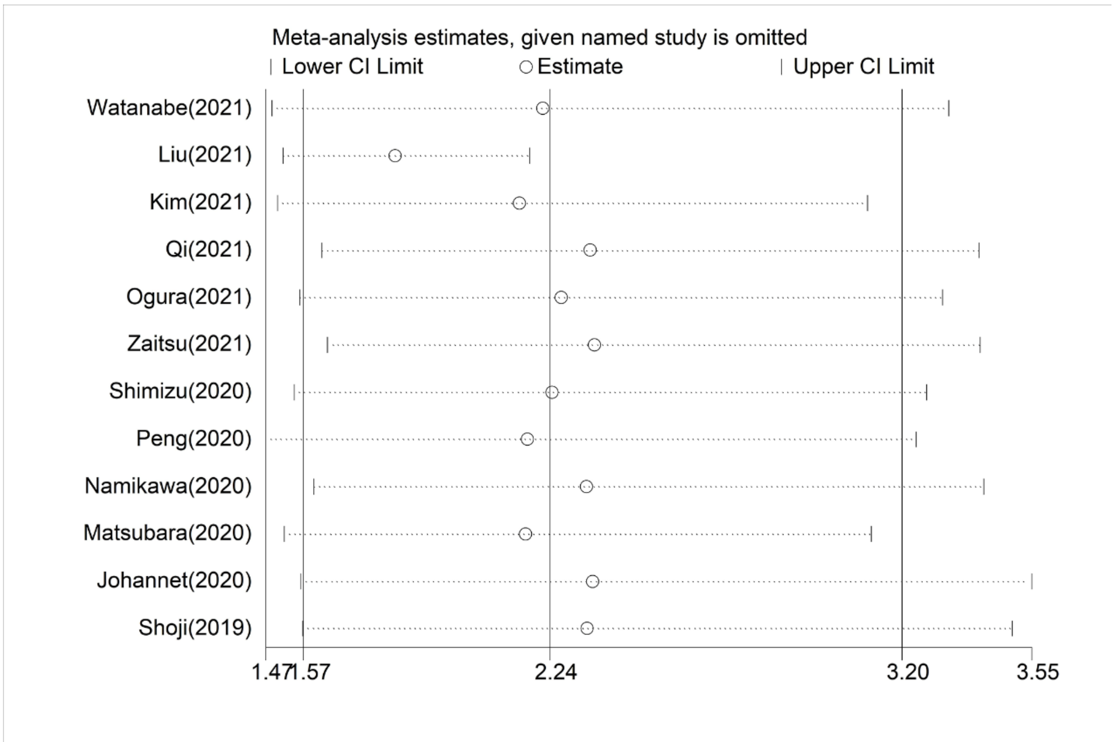

sFig.10 Metaninf test of studies included in meta-analysis of PNI for OS

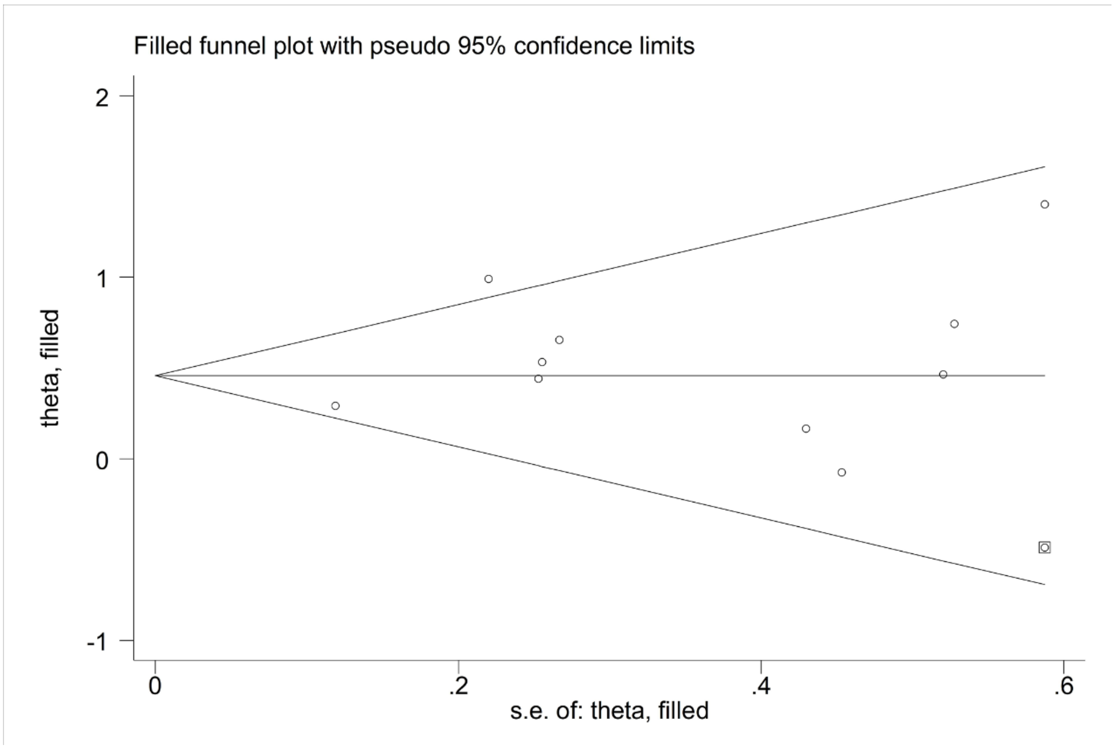

sFig.11 Metatrim test of studies included in meta-analysis of PNI for PFS (new pHR = 1.580, 95%CI = 1.580−1.353)

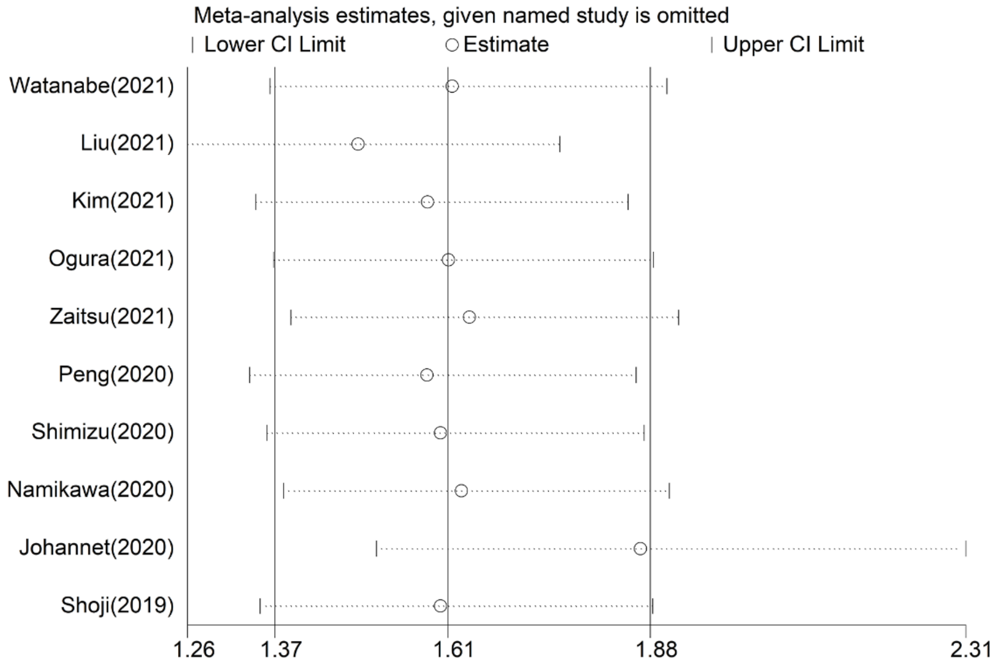

sFig.12 Metaninf test of studies included in meta-analysis of PNI for PFS
